# Low-dose oral imatinib in the treatment of systemic sclerosis interstitial lung disease unresponsive to cyclophosphamide: a phase II pilot study

**DOI:** 10.1186/ar4606

**Published:** 2014-07-08

**Authors:** Paolo Fraticelli, Barbara Gabrielli, Giovanni Pomponio, Gabriele Valentini, Silvia Bosello, Piersandro Riboldi, Maria Gerosa, Paola Faggioli, Roberto Giacomelli, Nicoletta Del Papa, Roberto Gerli, Claudio Lunardi, Stefano Bombardieri, Walter Malorni, Angelo Corvetta, Gianluca Moroncini, Armando Gabrielli

**Affiliations:** 1 Clinica Medica, Department of Internal Medicine, Ospedali Riuniti Ancona, Via Conca 71, 60020 Ancona, Italy; 2 Rheumatology unit, Second University of Naples, Via Pansini, 5 80131 Naples, Italy; 3 Division of Rheumatology, Catholic University, Via G. Moscati, 31 - Rome, 00168, Italy; 4 Allergy, Clinical Immunology & Rheumatology Unit, IRCCS Istituto Auxologico Italiano, Department of Internal Medicine, University of Milan, Piazzale Brescia, 20, 20149 Milan, Italy; 5 Department of Clinical Sciences and Community Health, University of Milan, Via Francesco Sforza 35, 20122 Milan, Italy; 6 Internal Medicine, Ospedale Nuovo di Legnano, Via Papa Giovanni Paolo II, 20010 Legnano, Italy; 7 Rheumatology Unit, Department of Biotechnological and Applied Clinical Science, University of L'Aquila, Piazzale Salvatore Tommasi 1, 67100 Coppito, L'Aquila, Italy; 8 Rheumatology Unit, Ospedale “G. Pini”, Piazza Cardinale Andrea Ferrari, 1, 20122 Milan, Italy; 9 Rheumatology Unit, Department of Medicine, University of Perugia, Via Enrico Dal Pozzo - padiglione X, 06122 Perugia, Italy; 10 Clinical Immunology and Rheumatology, Department of Medicine, University of Verona, P. le L.A. Scuro 10, 37134 Verona, Italy; 11 Rheumatology Unit, Department of Clinical and Experimental Medicine, University of Pisa, Via Roma 67, 56126 Pisa, Italy; 12 Department of Drug Therapy and Medicine Evaluation, Istituto Superiore di Sanità, Viale Regina Elena 299, 00161 Rome, Italy; 13 Internal Medicine and Rheumatology, Ospedale degli Infermi, Viale Luigi Settembrini, 2, 47923 Rimini, Italy; 14 Department of Clinical and Molecular Sciences, Università Politecnica delle Marche, Via Tronto 10/A, 60100 Ancona, Italy

## Abstract

**Introduction:**

Pulmonary involvement represents a major cause of death of systemic sclerosis (SSc) patients. Recent data suggest that tyrosine kinase inhibitors, such as imatinib, may be a therapeutic option for SSc patients. However, preliminary published clinical trials were inconclusive about imatinib efficacy and showed side effects. The purpose of this study was to verify efficacy and tolerability of low-dose imatinib on interstitial lung disease in a cohort of SSc patients unresponsive to cyclophosphamide therapy.

**Methods:**

Thirty consecutive SSc patients with active pulmonary involvement, unresponsive to cyclophosphamide, were treated with imatinib 200 mg/day for 6 months followed by a 6-month follow-up. A “good response” was defined as an increase of forced vital capacity (FVC) by more of 15% and/or increase of diffusing capacity of carbon monoxide (DL_CO)_ >15% and PaO_2_ > 90% of initial value and high-resolution computed tomography (HRCT)-scan pattern unchanged or improved.

**Results:**

Twenty-six patients completed the study. Three patients died and one patient was lost to follow-up. Four patients (15.32%) had a good response, 7 worsened and 15 had a stabilized lung disease. Overall, 19 (73.07%) patients had an improved or stabilized lung disease. After a 6-month follow-up, 12 (54.5%) of the 22 patients showed an improved or stabilized lung disease.

**Conclusions:**

Lung function was stabilized in a large proportion of patients unresponsive to cyclophosphamide therapy and a beneficial outcome emerged from the analysis of HRCT lung scans. There was no significant improvement of skin involvement, and the low dose was well tolerated. These data provide useful suggestions to design future randomized clinical trials for SSc therapeutics.

**Trial registration:**

ClinicalTrials.gov NCT00573326. Registered 13 December 2007.

## Introduction

Systemic sclerosis (SSc; scleroderma) is a rare, multisystem connective tissue disease characterized by widespread microvascular lesions and increased deposition of extracellular matrix components in the skin and internal organs [1].

Systemic sclerosis interstitial lung disease (SSc-ILD) and pulmonary arterial hypertension are a major cause of scleroderma-related mortality, accounting for over 60% of all deaths [2]. Depending on the diagnostic method used, interstitial lung disease is present in from 40 to 90% of SSc patients as bilateral basal alveolitis evolving to diffuse fibrosis and restrictive respiratory failure [3-6]. The pathogenesis of SSc-ILD is unknown and is probably the result of a combination of inflammatory and immunologic mechanisms, which lead to destructive and fibrotic lesions. Available therapies for the treatment of SSc-ILD are scarce and of limited benefit.

Imatinib mesylate (Gleevec; Novartis Europharm Ltd, Wimblehurst Road, Horsham, West Sussex RH12 4AB, UK) has been approved for the therapy of chronic myelogenous leukemia and gastrointestinal stromal tumors [7]. Interest in use of imatinib mesylate in SSc lies in its ability to interfere with the tyrosine kinases downstream of the receptors for transforming growth factor beta and platelet-derived growth factor, which are considered key signaling molecules in the pathogenesis of SSc fibrosis [8,9].

Six clinical pilot trials in SSc patients have been published, with a focus on the safety and effectiveness of imatinib mesylate in the treatment of both cutaneous and pulmonary involvement [10-15]. The results have been controversial with regard to efficacy and all studies were generally characterized by a poor tolerability.

In view of these discordant data, we designed a multicenter, prospective, open-label trial with the following characteristics: only SSc patients with active lung involvement, despite previous therapy with cyclophosphamide, were enrolled; a single dose of 200 mg/day imatinib mesylate was used; the study was structured according to Simon’s optimal two-stage design [16,17]; and the goal was to assess the effect on lung fibrosis and alveolitis, as well as the tolerability of this regimen as a therapy for SSc-ILD.

## Methods

### Study design

To evaluate the effect of the treatment with imatinib mesylate on SSc-ILD, the study was structured according to Simon’s optimal two-stage design [16], a strategy devised for phase II studies to limit the number of subjects who will undergo inefficacious therapy, consisting of two subsequent enrolment phases [16,17]. Ten patients (variable: *n*_1_) entered the first stage of the trial. Enrolment would have been stopped and the drug rejected if <10% of the patients (variable: *P*_0_) had met the primary end point at 6 months. Conversely, the study would have been continued until 30 patients were enrolled in total (variable: *n*). The drug would have been rejected in the case of a positive response rate in <30% (variable: *P*_1_) of the patients completing the study.

The values of variables *n*_1_, *n*, *P*_0_, and *P*_1_ were set to obtain a probability ≤0.05 of accepting a drug worse than *P*_0_ after the first stage and a probability ≤0.2 of rejecting a drug better than *P*_1_ at the end of the study.

### Study population

From February 2009 to June 2011, 72 consecutive patients with SSc and active pulmonary involvement that had not improved after cyclophosphamide treatment were screened for inclusion/exclusion criteria at 12 Italian centers. The reasons for exclusion were all registered. All SSc patients fulfilled the American College of Rheumatology preliminary criteria for the classification of SSc [18]. The protocol, patient information sheet and consent form were approved by the Ethics Committee of each participating centre (see Acknowledgements for specific names of the ethical bodies). The study was conducted in accordance with the Declaration of Helsinki in its fifth edition (2000). Written informed consent was obtained from all patients.

The diagnosis of active lung involvement required the presence of grade 2 exertional dyspnea according to the Mahler Baseline Dyspnea Index [19] plus interstitial alveolitis assessed by high-resolution computed tomography (HRCT) scan (ground-glass opacifications involving at least two lung segments) or neutrophilic (>3%) or eosinophilic (>2%) alveolitis detected by bronchoalveolar lavage. SSc patients were defined resistant to conventional immunosuppressive treatment if the forced vital capacity (FVC) and/or diffusing lung capacity of carbon monoxide (DL_CO_) had not increased by more than 15% after a cumulative dosage of ≥6 g cyclophosphamide. SSc patients were considered resistant to the treatment also in the event of a partial pressure of oxygen in arterial blood decrease ≥15% compared with baseline values. Evidence of active lung involvement before cyclophosphamide therapy initiation had to be documented.

Inclusion criteria were: SSc as defined above; presence of active interstitial alveolitis; resistance to conventional immunosuppressive treatment; age 18 to 80 years; ability to give informed consent; and use of an acceptable method of birth control (if applicable). Pregnancy had to be ruled out before the beginning of the study.

Exclusion criteria were: severe pulmonary fibrosis, defined as interstitial fibrosis (honeycombing pattern) involving ≥50% of the lung (assessed by HRCT scan); connective tissue diseases other than SSc; smoking habit; pregnancy or lactation; evidence of hepatic B virus or hepatic C virus infection; severe anemia (hemoglobin ≤ 8 g/dl); hepatic disease (alanine transaminase or alkaline phosphatase >1.5-fold above normal levels); moderate or severe renal failure (creatinine clearance <59 ml/minute); severe heart failure or ejection fraction ≤35% measured by echocardiography; or evidence of thyroid disease.

### Treatment

Eligible patients received oral imatinib mesylate (Gleevec; Novartis Europharm Ltd) 200 mg (two tablets) once a day, after breakfast, for 6 months. The drug was supplied every 3 months during the followup visits. The drug tablets were counted and returned if they had not been used by patients for any reason. Immunosuppressive medications were not allowed within 3 months prior to initiation of imatinib. Cyclophosphamide had been stopped between 3 and 48 months (mean interval 14.96 months) before the start of the study. A stable dose of oral prednisone (≤15 mg/day) as well as calcium channel blockers or iloprost were allowed. Every other symptomatic therapy was permitted if not interfering with imatinib. Patients did not receive immunosuppressive drugs in the 6 months following treatment with imatinib.

### Assessments

The study was open label for the patients and the physicians directly involved in patient clinical evaluation.

Variables measured every 3 months included electrocardiography, routine blood and urine tests (urea, electrolytes, serum glucose, liver function tests urinalysis, resting arterial blood gases, complete blood cell count, and 24-hour proteinuria), thyroid function tests, and creatine kinase.

Pulmonary function tests and single-breath diffusing capacity were performed in accordance with recommended standards [20,21]. Physicians performing lung function tests were not aware of being in an experimental setting and were blind to the treatment.

Results were expressed as liters (FVC) and ml/mmHg/minute (DLco). Percentage variation between values recorded at baseline and 6 months was calculated as:

$$\Delta =\left(T6/T0\times 100\right)–100$$

Pulmonary function tests and an HRCT scan were performed at baseline, 6 months after the beginning of treatment, and at 12 months (i.e. after a 6-month follow-up) in the absence of clinical events that would interfere with their interpretation.

Assessment of the HRCT scan pattern consisted of sequential acquisition of 1 mm scans, spaced at 10 mm, extending from the lung apices to below the costo-phrenic angles. Images were reconstructed with a high spatial frequency algorithm for lung analysis and were viewed with lung window settings. Lung evaluation was performed at suspended end-inspiratory volume and examination was carried out with patients in the prone position to avoid lung opacities in the posterior lung. No intravenous contrast material was used. Twenty bronchopulmonary segments were evaluated and a segment was considered involved if ≥50% had a ground-glass appearance [22].

HRCT scans were independently analyzed in a random order by three radiologists who were not informed of whether scans had been obtained before or after therapy. Disagreements were resolved through collegial discussion or by majority rule. Pulmonary function tests were also evaluated blindly irrespective of the treatment.

The extent of skin involvement was assessed by trained physicians using the modified Rodnan skin score (mRSS), as described elsewhere [23]. *Ad hoc* training was performed before the start of the study to limit intra-observer and inter-observer variation less than 5%.

### Outcomes

The first primary outcome was improvement of the pulmonary involvement. Improvement was defined as an increase of FVC >15% and/or increase of DL_CO_ >15% *and* partial pressure of oxygen in arterial blood >90% of initial value *and* HRCT scan pattern defined as unchanged or improved. HRCT was considered improved if there was disappearance of ground-glass opacities in at least two lung segments. The patient was considered worsened if FVC or DL_CO_ decreased >15% or if HRCT was worsened. Change of FVC and DL_CO_ between −15 and +15% with an unchanged/improved HRCT was considered evidence of stabilized disease. The second primary outcome was drug tolerability.

Secondary outcomes were a reduction of skin thickness evaluated by the mRSS and patient physical and emotional wellness assessed by the Medical Outcomes Short Form-36 score, Health Assessment Questionnaire, and visual analogue scale for global wellness [24].

### Study oversight

All consecutive SSc patients with active lung involvement were screened by the participating centers for inclusion/exclusion criteria and identified with a PIN to warrant privacy and anonymity.

A trained investigator from the Ancona Centre was the internal quality controller of the study. All centers were subject to direct and indirect controls (drugs’ accountability, blinding procedures, control of case record forms). After every quality control, an audit concerning the wrong procedures was performed.

Adverse events were managed and classified as light, moderate, and severe and as related, probably related, and unrelated to the study drug, according to the commonly accepted guidelines [25].

### Statistical analysis

Since the data were not distributed normally, they were expressed as median (25th to 75th percentile ranges) unless otherwise stated. Comparisons were made using the nonparametric Wilcoxon test. Differences were considered statistically significant if *P* ≤ 0.05 (two-tailed).

## Results

**Figure 1:**
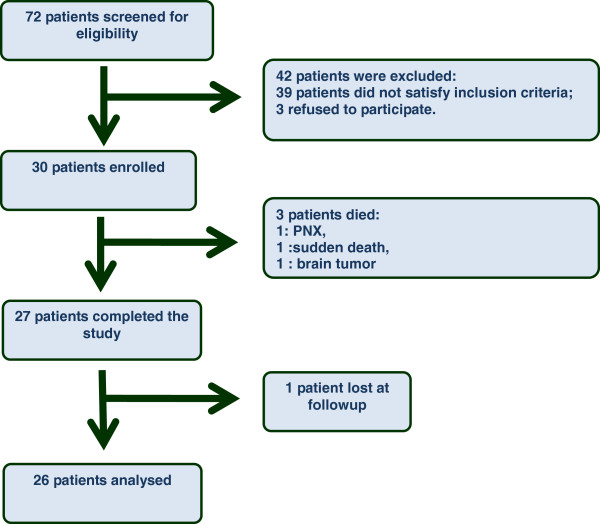
**Trial profile.** Six-month data were obtained on all but four patients. At 12-month visit, pulmonary function tests were available in 22 patients. PNX, pneumothorax.

The trial profile is outlined in Figure 1. Seventy-two patients were screened, and 42 were excluded: 39 for not satisfying inclusion criteria (Figure 1) and three for lack of consent. Pretreatment demographic, clinical, and immunological features of the 30 enrolled patients are summarized in Table 1. Median age was 51 (41.75 to 62) years and median disease duration (defined as the time from the onset of the first non-Raynaud’s phenomenon clinical manifestation) was 3 years [1-6]. Nine patients (30%) were male and 21 patients (70%) were female, all Caucasian. Fourteen patients (46%) had limited cutaneous SSc and 16 patients (53.3%) had diffuse cutaneous SSc. The majority of the patients (93.3%) were anti-topoisomerase-I-positive; one patient was anti-centromere-positive and one patient was Antinuclear antibodies-positive but lacked specific SSc autoantibodies. In the whole group, median systolic pulmonary artery pressure detected by echocardiography was 30 (28.5 to 36.5) mmHg. After 12 months, 26 patients completed the study, three patients died and one patient, who did not complete the 6-month examination, was excluded from the final analysis. The data therefore refer to 26 patients.

**Table 1:** Baseline demographic, clinical and immunological features of the study population

Male/female	9 (30%)/21 (70%)
Age (years)	51 (41.75 to 62)
Disease duration (years)	3 (1 to 6)
Anti-DNA topoisomerase I/anti-centromere antibodies	28 (93.3%)/1 (3.3%)
Systemic sclerosis, lcSSc/dcSSc	14 (46%)/16 (53.3%)
Pulmonary function tests	
FVC% predicted	76.5 (63.23 to 89.30)
TLC% predicted	75 (65 to 86)
DLco% predicted	43 (37 to 54)
High-resolution computed tomography	
Ground-glass	10 (6 to 13.75)
Honeycombing	10.5 (6.25 to 16.25)
Modified Rodnan skin score	10.5 (5.25 to 20)
Health Assessment Questionnaire	0.375 (0 to 1)

Data presented as number of patients (%) or median (25th to 75th percentile). dcSSc, diffuse cutaneous systemic sclerosis; DLco, diffusion lung capacity of carbon monoxide; FVC, forced vital capacity; lcSSc, limited cutaneous systemic sclerosis; TLC, total lung capacity.

### Efficacy

After 6 months of treatment with imatinib, lung involvement improved in four of 26 patients (15.32%), worsened in seven patients (26.92%), and remained stable in 15 patients (57.69%). Nineteen (73.07%) patients thus had improved or stabilized lung disease (Table 2).

**Table 2:** Pulmonary function tests and mRSS at baseline and 6 months, and HRCT outcome in 26 systemic sclerosis patients treated with imatinib

**Patient (PIN)**	**FVC (l) T0**	**FVC (l) T6**	**ΔFVC (T0 – T6) (%)**	**DLco (ml/mmHg/minute) T0**	**DLco (ml/mmHg/minute) T6**	**ΔDLco (T0 – T6) (%)**	**HRCT**^**a **^**T0**	**HRCT**^**a **^**T6**	**HRCT (evaluation at T6)**	**mRSS T0**	**mRSS T6**	**ΔmRSS (T0 – T6) (%)**	**Months from the end of CYC/cumulative dose of CYC/other therapies**
**86**	**3.23**	**3.84**	**−5.88**	**8.45**	**10.78**	**27.57**	**19/18**	**4/18**	**Improved**	**4**	**3**	**−25**	**21/10 g/Aza**
**509**	**3.36**	**2.71**	**–19.3**	**10.64**	**14.05**	**32.05**	**6/2**	**4/2**	**Improved**	**24**	**31**	**29.16**	**48/7.5 g/Mtx**
**642**	**1.53**	**1.57**	**2.61**	**8**	**10**	**25**	**9/9**	**6/9**	**Improved**	**5**	**6**	**20**	**11/70 g**
**649**	**1.79**	**1.83**	**2.23**	**8.44**	**9.54**	**19.62**	**6/8**	**7/9**	**Unchanged**	**3**	**3**	**0**	**3/10 g/CyA**
18	2.35	2,31	–1.7	18.99	19.92	4.9	6/8	3/8	Improved	0	0	0	42/13.5 g/Aza
115	2.69	2.45	–8.92	16.1	17	6.83	4/2	4/2	Unchanged	20	20	0	12/18 g/Aza, Rtx
205	1.52	1.43	–5.92	4.9	5	2.04	6/20	0/20	Improved	5	6	29.16	22/21 g
214	3.16	3,16	0	12.9	14	8.53	10/8	1/9	Improved	29	25	−13.79	9/6 g
219	1.97	1.83	−7.1	8.5	8.1	−4,7	20/20	20/20	Unchanged	10	16	60	36/6 g/Aza
297	2,19	2.24	2.28	7.2	7.49	4.02	13/7	12/7	Unchanged	22	17	−22.72	20/6 g
304	2.41	2.56	6.22	15.1	14.4	−4.63	12/3	11/3	Unchanged	11	9	−18.18	3/6 g
371	3.14	3.56	13.37	17.8	19.7	10.67	6/8	0/8	Improved	6	6	0	4/8.2 g
432	1.77	1.82	2.82	5.31	5.77	8.66	14/17	13/17	Unchanged	9	9	0	8/9 g
482	1.55	1.43	−7.74	7.68	7.39	−3.78	11/14	1/14	Improved	5	4	−20	7/9 g
522	3.20	3.08	−3.75	13.18	13.2	0.15	12/6	4/5	Improved	28	31	10.71	10/9 g
586	2.21	2.42	9.5	8.6	9.1	5.81	6/12	5/12	Unchanged	28	30	7.14	3/6 g
696	3.29	3.27	−0.6	14.5	14.19	2.76	11/6	7/7	Improved	8	10	25	15/7,2 g
763	3.81	3.75	−1.5	16.4	16.9	3.05	14/13	8/13	Improved	17	20	17.64	15/6 g/Rtx
801	2.09	2.13	1.9	10.5	11.5	9.52	16/12	8/13	Improved	15	11	26.66	3/7,2 g
*144*	*3.56*	*3.57*	*0.28*	*9.65*	*6.16*	*–36.16*	*9/14*	*4/16*	*Worsened*	*15*	*15*	*0*	*6/10 g*
*358*	*2.24*	*1.81*	*–19.2*	*8.30*	*7.6*	*–8.63*	*8/14*	*4/14*	*Improved*	*14*	*24*	*71.42*	*12/38 g/Aza*
*459*	*2.44*	*1.97*	*–19.26*	*10.4*	*9.9*	*–4.81*	*14/20*	*14/20*	*Unchanged*	*25*	*27*	*8*	*15/9 g*
*534*	*3.26*	*3.01*	*−7.6*	*15.12*	*14.93*	*−1.26*	*5/4*	*10/12*	*Worsened*	*0*	*0*	*0*	*28/10 g/Aza*
*552*	*1.95*	*1.95*	*0*	*8.98*	*7.86*	*−11.36*	*18/18*	*20/20*	*Worsened*	*28*	*31*	*10.71*	*8/20 g/Aza, MMF*
*706*	*1.86*	*1.7*	*−8.6*	*6.21*	*3.55*	*−42.83*	*6/20*	*6/20*	*Unchanged*	*10*	*10*	*0*	*7/6 g/Aza*
*789*	*2.04*	*2.12*	*3.9*	*10.7*	*10.4*	*−2.8*	*10/4*	*10/10*	*Worsened*	*7*	*10*	*42.85*	*21/7.2 g*

Bold data indicate that patients responders: Roman data indicate that patients stabilized; italic data indicate that patients worsened. Aza, azathioprine; CyA, cyclosporine A; CYC, cyclophosphamide; DLco, diffusion lung capacity of carbon monoxide; FVC, forced vital capacity; HRCT, high-resolution computed tomography; Mtx, methotrexate; MMF, mycofenomycophenolate mophetil; mRSS, modified Rodnan skin score; Rtx, rituximab; T0, baseline; T6, 6-month visit; Δ = (T6/T0 × 100) – 100. ^a^Number of segments with ground-glass/honeycombing.

Compared with baseline, three of the four patients classified as improved showed evidence of HRCT improvement, while HRCT was unchanged in the remaining patient (Table 2). In one out of the four patients with interstitial lung disease improved by imatinib (Patient 509), the discrepancy between the improved DLco and the worsened FVC is probably to be ascribed to chest wall restriction secondary to the increase of mRSS. In the subgroup of 15 patients with stabilized disease, HRCT was improved in nine patients and unchanged in six patients. Interestingly, pulmonary functional tests lagged behind improvement in HRCT in all patients.

Three of the seven patients who were classified as worsened had an improved or unchanged HRCT (Table 2).

**Figure 2:**
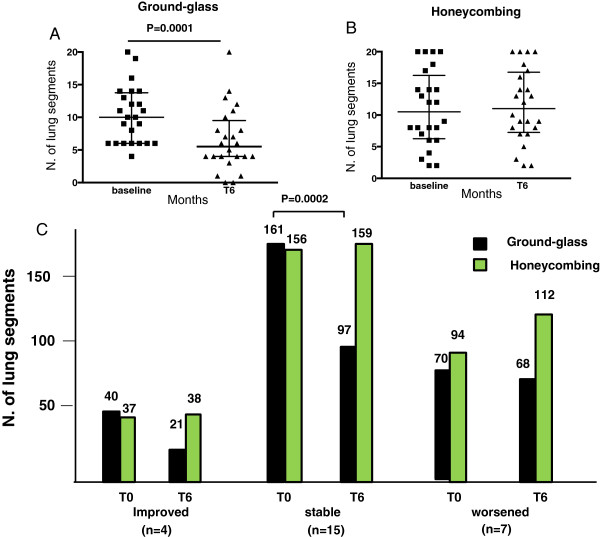
**Number of lung segments with high-resolution computed tomography ground-glass opacities and honeycombing in all patients at baseline and at 6 months.** Number of lung segments with high-resolution computed tomography ground-glass opacities **(A)** and honeycombing **(B)** in all 26 patients at baseline and at 6 months (T6), and in the subgroups of patients classified as improved, stabilized, and worsened after 6 months of treatment with imatinib **(C)**. **(A)**, **(B)** Data expressed as median with interquartile ranges. **(C)** Data expressed as absolute numbers of lung segments.

Figure 2A shows that after 6 months of treatment with imatinib the median number of lung segments with ground-glass opacities was significantly reduced compared with baseline (5.5, 25th to 75th percentile ranges 4.0 to 9.50 vs. 10, 25th to 75th percentile ranges 6.0 to 13.75) (*P* = 0.0001). No difference was detected with regard to the number of lung segments with honeycombing (10.5, 25th to 75th percentile ranges 6.25 to 16.25 at T0; and 11, 25th to 75th percentile ranges 7.25 to 16.0 at T6) (*P* = NS) (Figure 2B). Patients classified as improved or stabilized showed a relevant reduction in the number of lung segments with ground-glass opacities (Figure 2C). This was statistically significant in the subgroup of stable patients (*P* = 0.0002). In the improved subgroup, the number of patients was too small for the difference to be statistically significant.

In the subgroup of 19 patients with either improved or stabilized lung disease, one patient had disappearance of ground-glass opacities in two segments, and 11 patients in more than two segments (Table 2).

At month 12, 6 months after the end of treatment, 12 of the 22 patients (54.5%) whose pulmonary function tests were available for analysis had an improved or stabilized disease compared with baseline (data not shown).

With regard to the secondary outcomes, no significant improvement of the mRSS was noticed (data not shown). Wellness, physical activities, and quality of life – assessed by the visual analogue score, Health Assessment Questionnaire, and the Short Form-36 questionnaires respectively – did not show any significant improvement except for the emotional role of the Short Form-36 score (data not shown).

### Adverse events

Serious adverse events requiring drug discontinuation were registered in three cases (10%) (Table 3).

**Table 3:** Serious adverse events

**Patient PIN**	**Event**	**Duration of experimental therapy**	**Attribution**
178	Pneumothorax due to central line insertion. Death	5 days	Not related
384	Malignancy. Death	139 days	Probably not related
663	Sudden death	11 days	Not related
459	Severe pneumonia with respiratory failure	6 months (17 days after the last dose of imatinib)	Unlikely related

One patient (Patient 178) died as a consequence of pneumothorax occurring after central line positioning. The patient was in poor clinical condition, and severely malnourished due to malabsorption. Therapy with imatinib was discontinued 5 days after the start. The adverse event in this patient was judged to be not related to imatinib.

In a second patient (Patient 384) a neuroendocrine tumor localized to the skull, meninges, and brain was diagnosed 139 days after the start of imatinib. The primary tumor was ascribed to a pelvic mass with the same histopathological features. Imatinib was discontinued and chemotherapy and radiotherapy were initiated. The patient died 44 days later. The tumor was probably present at the time of enrolment and had not been detected by the screening procedures. This adverse event was judged as probably not related to imatinib.

Sudden death occurred in a third patient (Patient 663). She was found dead in a primary care hospital where she had been referred for weakness, lower limb edema, and diarrhea, which had started a few days after colchicine (1 mg/day) had been administered for condrocalcinosis. An autopsy was not performed. The patient had been on imatinib for 11 days. The adverse event in this patient was judged to be not related to imatinib.

A fourth patient (Patient 459) developed severe pneumonia requiring noninvasive ventilation 17 days after completing the 6-month therapy, The patient had advanced lung fibrosis and a long history of steroid treatment. The patient completely recovered. The adverse event in this patient was judged unlikely to be related to imatinib.

Overall adverse events were present in less than 20% of the patients, mainly transient and well tolerated, without subsequent withdrawal or dose adjustment of the experimental drug. The most common adverse events were lower limb edema, cough, and infections (Table 4).

**Table 4:** Adverse events in 26 systemic sclerosis patients treated with imatinib

**Adverse event**	**Number of patients**	**%**
Lower limb edema	5	19.2
Cough	5	19.2
Infections	5	19.2
Urticaria/rash	4	15.3
Arthritis	2	7.6
Transient creatine kinase elevation	1	3.8
Diarrhea	1	3.8
Thrombophlebitis	1	3.8
Others	4	15.3

## Discussion

Using strict enrolment and assessment criteria, only 15.3% of SSc patients with active interstitial lung disease could be classified as improved after 6 months of treatment with low-dose imatinib. This result fell short of the 30% rate target.

However, although the comparison is very problematic for the reasons stated below, the results shown here are encouraging when compared with those of Tashkin and colleagues. Their study showed that improvement of FVC between 10 and 15% was obtained in 5% and <5% of SSc patients, respectively, after 12 months of oral cyclophosphamide or placebo [26].

In the present study, the failure to meet the primary end point could be ascribed to the clinical features of the patients enrolled in this study, who had severe lung disease as manifested by the lack of improvement after intensive treatment with cyclophosphamide, and in some patients with other immunosuppressive drugs also, and to the target of ≥15% increase in pulmonary functional tests (FVC and/or DLco) plus unchanged or improved lung HRCT as evidence of a positive outcome. The cumulative percentage of responder and stabilized patients (73.07%) was high, but these data must be interpreted with caution. First, a carryover effect of previous immunosuppressive therapy cannot be ruled out, although worsening occurred in patients who had received an immunosuppressive therapeutic regimen like that of improved/stabilized patients. Furthermore, it is worth noting that this was an open trial without a control group representative of the natural progression of the disease, and to our knowledge there are no data about the rate of progression of lung alveolitis in patients who had failed cyclophosphamide therapy.

Some patients with active SSc-ILD, however, may benefit from imatinib treatment, especially those with HRCT of the lung characterized by ground-glass (Figure 2). Identification of these patients is crucial, and in this respect it is reasonable to suggest that: future trials with tyrosine kinase inhibitors should include patients with less advanced lung disease – treating patients at an earlier stage could be more rewarding, as suggested by the lack of effect of imatinib on established fibrosis; longer treatment should also be taken into consideration, since pulmonary function tests lagged behind the improvement documented by HRTC; fixed doses of the drug have so far been used in all clinical trials that have explored imatinib, but alternative regimens with a tailored dose should be evaluated to maximize the benefit/adverse effects ratio; patients should be selected based on the level of activation of platelet-derived growth factor receptors in lung tissue [27]; and other tyrosine kinase inhibitors may have more efficacious therapeutic effects.

Not significant improvement was seen in the extent of skin fibrosis, probably as a result of enrolment of patients with minimal skin involvement.

The main difference between this and previous imatinib trials is the lower dose employed and the enrollment of patients who had failed immunosuppressive therapy. In their pilot study, Khanna and colleagues found a statistically nonsignificant improvement in the predicted FVC%, and radiological findings were not included in their evaluation. Eight out of 20 SSc patients who received up to 600 mg imatinib/day did not complete the study either because of adverse events (seven patients) or because they were lost to follow-up (one patient) [12]. While the study of Pope and colleagues was stopped early because of poor tolerability of imatinib (200 mg twice a day) [10], the open-label single-arm clinical trial of Spiera and colleagues demonstrated improvement of mRSS after administration of imatinib 400 mg daily [11]. Twenty-four of 30 patients completed 12 months of therapy and their predicted FVC improved by 6.4% (*P* = 0.008). No data regarding HRCT were provided. In the study by Distler and colleagues, imatinib (up to 600 mg daily) had no significant effect on mRSS in a group of patients with early diffuse SSc [13]. Treatment was characterized by poor tolerability and adverse events. Finally, no efficacy on mRSS was demonstrated by Prey and colleagues in a randomized double-blinded controlled study using 400 mg daily in a mixed population of patients with morphea and SSc patients with extensive skin involvement [15].

This trial raises some speculation about the mechanisms of action of imatinib. The disappearance of ground-glass opacities implies an immunomodulatory mechanism, which is probably based on the competitive inhibition of distinct tyrosine kinases by imatinib. Besides platelet-derived growth factor receptor α/β, the nonreceptor Abelson tyrosine kinase (c-abl) [28], stem cell factor receptor c-KIT [28], macrophage colony-stimulating factor receptor (c-fms) [29] and lymphocyte-associated kinase (lck) [30] – which are all involved in development, activation, proliferation and function of immune cells – are inhibited by imatinib. In this respect, imatinib has been investigated in several models of kidney disease [31] and shown to ameliorate immunologic and fibrotic features. Furthermore, imatinib 100 mg/kg suppressed the progression and attenuated the severity of established experimental encephalomyelitis, a mouse model of multiple sclerosis [32], of autoimmune arthritis [33], and of autoimmune diabetes in mice [34]. One can thus speculate that inhibition of immune response may contribute to halting the progression of the fibrotic mechanisms regardless of the pathogenesis.

The low dose used was probably responsible for the negligible side effects observed in this study. This differs remarkably from the results of the other published trials, which were characterized by the poor tolerability of the treatment due to the higher doses employed [10-13].

With regard to the adverse events in our study, we experienced three deaths and one serious adverse event. The three patients who died had a severe and advanced form of disease. One patient had fatal complications after wrong positioning of a central line. A second patient was diagnosed with a neuroendocrine tumor localized to the skull, meninges, and brain after starting the experimental drug. In this case, imatinib was stopped immediately but despite prompt chemotherapy and radiotherapy the patient died after few weeks. The third patient, who was taking colchicine for condrocalcinosis, suffered a sudden death in a primary care hospital where she was referred for weakness, lower limb edema, and diarrhea. Unfortunately an autopsy was not performed. The last patient developed severe pneumonia requiring noninvasive ventilation but she had advanced fibrosis and had completed the therapy 17 days earlier. We think that two deaths are not related to the treatment and the third is unlikely to be related, and all were as a consequence of the severity of the disease.

## Conclusions

Low-dose imatinib stabilized pulmonary involvement in scleroderma patients who had not previously responded to cyclophosphamide. Side effects were negligible and the dosage employed was well tolerated. Together with most recently published pilot studies, these data make this drug attractive for future randomized clinical trials.

## Abbreviations

DLco: diffusing lung capacity of carbon monoxide; FVC: forced vital capacity; HRCT: high-resolution computed tomography; mRSS: modified Rodnan skin score; SSc: systemic sclerosis; SSc-ILD: systemic sclerosis interstitial lung disease.

## Competing interests

The authors declare that they have no competing interests

## Authors’ contributions

AG, PFr, GP, GV, and BG were responsible for study concept and design, modification of study design, and review and interpretation of data. AG and PFr were also responsible for drafting the manuscript. SB, PR, MG, and PFa provided modifications of study design and revisited the manuscript. RGi, NDP, RGe, CL, SB, WM, and AC, contributed to collection, analysis and interpretation of data and revisited the manuscript. GM contributed to collection, analysis and interpretation of data and revisited the manuscript. All authors read and approved the final manuscript.

## Authors’ information

Imatinib in Scleroderma Italian Study Group: Anna Maria Giammarioli, MD (Dipartimento di farmacologia, Istituto Superiore di sanità, Roma, Italy); Giovanna Cuomo MD, Michele Iudici MD (U.O di Reumatologia, Seconda Università di Napoli, Napoli, Italy); Elena Bartoloni MD (U.O. di Reumatologia, Dipartimento di Medicina, Università di Perugia, Perugia, Italy); Paola Cipriani MD (U.O. di Immunoreumatologia Università dell’Aquila, L’Aquila, Italy); Lisa Gabbriellini (U.O. Allergologia, Immunologia Clinica e Reumatologia, Istituto Auxologico Italiano, Milano, Italy); Silvia Svegliati PhD, Donatella Amico MD, Matteo Marcosignori MD, Martina Bonifazi MD, Simona Giori MD (U.O. Clinica Medica, Dipartimento di Medicina Interna, Ospedali Riuniti, Ancona, Italy).
